# Evaluation of tolerance to artificial gastroenteric juice and fermentation characteristics of *Lactobacillus* strains isolated from human

**DOI:** 10.1002/fsn3.2662

**Published:** 2021-12-10

**Authors:** Chen Liu, Fei Han, Lin Cong, Ting Sun, Bilege Menghe, Wenjun Liu

**Affiliations:** ^1^ Key Laboratory of Dairy Biotechnology and Engineering Ministry of Education of China Inner Mongolia Agricultural University Hohhot China; ^2^ Key Laboratory of Dairy Products Processing Ministry of Agriculture and Rural Affairs of China Inner Mongolia Agricultural University Hohhot China; ^3^ Inner Mongolia Key Laboratory of Dairy Biotechnology and Engineering Inner Mongolia Agricultural University Hohhot China

**Keywords:** bile tolerance, fermentation characteristics, intestinal *Lactobacillus*, isolation and identification, resistance to artificial gastroenteric juices

## Abstract

Fifty‐seven strains of *Lactobacillus* were isolated from fecal samples of healthy young people in Tibet, Xinjiang, and Inner Mongolia using pure culture methods. *Lactobacillus ruminis* and *Lactobacillus gasseri* were the dominant *Lactobacillus* species isolated from the intestinal microflora, accounting for 54.4% and 14.0% of the total isolates, respectively. Isolated strains were identified by 16S rRNA sequencing, and their tolerance to gastric acid and bile salt, and fermentation characteristics were evaluated. The results of experiments in vitro showed that nine of the isolated strains of *Lactobacillus* grew well at pH 3.0. After 11 h of incubation in artificial digestive juices, the isolated *L. plantarum* and the control strain *L. plantarum* P8 still had high survival rates. Most of the isolates and control isolates have strong tolerance to bile salts. The evaluation of fermentation characteristics indicated that the ability of the intestinal *Lactobacillus* to ferment skimmed milk was lower than that of the reference *L. plantarum* P8. In the process of storage, the viable count of screened isolates of human origin in fermented milk decreased to some extent, but remained above 7.01 ± 0.22 log CFU/ml, showing good storage characteristics.

## Graphical abstract

BLURB FOR ETOC:The pure culture method was used to isolate and identify the lactic acid bacteria in the sample. Strains with good characteristics were screened by simulating artificial gastrointestinal fluid. Finally, the fermentation characteristics of the strains were evaluated.

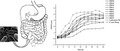

## 1 INTRODUCTION

The human intestinal microflora is composed of different microbes. To date, about 30 genera and more than 500 species and subspecies of bacteria have been isolated and identified; the vast majority of these are anaerobic bacteria (Min et al., 2004; Shang & Yu, 2012). There are more microbial cells within humans than there are human cells themselves, and 78.67% of those microbes are in the human gastrointestinal tract where they play a vital role in maintaining healthy homeostasis (Luckey, 1972). Intestinal microorganisms contribute to human health in the same way as an organ and so some scholars describe the metabolism of the microflora in the intestine as a hidden organ in the human body (Sekirov et al., 2010; Zhao & Shen, 2010). Human intestinal microflora was affected by many factors, such as geographical environment, eating habits, lifestyle, and age. Xinjiang, Tibet, and Inner Mongolia are the gathering places of nomads, following the ancient and traditional way of living. At the same time, these areas are located in the frontier and have a unique climate. With the growth and development of human body, the structure of human intestinal microflora is relatively stable, so we studied the intestinal microflora of young people in this area.

The term “probiotic” refers to microorganisms that benefit the host when administered in appropriate quantities (Hotel, 2001; Hill et al., 2014; Roberfroid, 2000). The best probiotics are of human origin that is safe and does not harbor genes associated with antibiotic resistance, pathogenicity, or virulence factors (Yasmin et al., 2020). At the same time, they must be able to survive under intestinal conditions (e.g., acid pH, enzymes, and bile salts) (Wang et al., 2009). It must also be demonstrated that the activity, viability, growth, and efficacy of probiotics are maintained in technical treatments (Plaza‐Díaz et al. 2017, 2018). Probiotics include microorganisms, most of which are similar to the beneficial bacteria naturally produced in the human intestine. To date, probiotics have been used in the treatment of a variety of gastrointestinal diseases and have been widely studied; the most studied species are from the genera *Lactobacillus* and *Bifidobacterium*, and also yeast (Wilkins & Sequoia, 2017). As one of the main probiotics, *Lactobacillus* species are also extremely important microflora in the intestine and their ability to survive in the gut environment is of great significance to host’s health.  

*Lactobacillus* species are important in intestinal homeostasis throughout the host's life. They can help correct imbalances in the intestinal microflora, thereby restoring and improving host’s health. Clinical studies have confirmed that probiotics are effective against gastrointestinal disorders, for example, irritable bowel syndrome (Zhang et al., 2016), *Helicobacter pylori* (Zheng et al., 2013), inflammatory bowel disease (Saez‐Lara et al., 2015), and diarrhea (McFarland, 2007); allergic diseases for example atopic dermatitis (Rather et al., 2016); in treating obesity (Sharafedtinov et al., 2013), insulin resistance syndrome (Rajkumar et al., 2014), type 2 diabetes and non‐alcoholic fatty liver disease (Sáez‐Lara et al. 2016; Buss et al., 2014), and the side effects of cancer (Redman et al., 2014). Positive effects of probiotics on human health can also be achieved through immune regulation (Hevia et al., 2015).

*Lactobacillus* is also commonly used in the production of fermented dairy products. In the food industry and dairy processing industry, it is used as a starter in the production of yogurt and cheese. In recent years, the use of lactic acid bacteria, particularly *Lactobacillus* species, has extended from food production into microecological preparations for use as health products. *Lactobacillus* has a very important role and economic value in our daily life. For this reason, there has been much research on the isolation, screening, metabolism, physiology, and genomics of intestinal *Lactobacillus* species.

In this study, the pure culture method was used to isolate and identify the *Lactobacillus* from the intestinal tracts of healthy young people in Tibet, Inner Mongolia, and Xinjiang. Preliminary evaluations were made on the acid and bile salt resistance and fermentation characteristics of the intestinal isolates of *Lactobacillus*. The experimental results may provide basic data for the screening and the evaluation of probiotic *Lactobacillus* and provide theoretical guidance for the industrial production of *Lactobacillus*.

## 2 MATERIALS AND METHODS

### 2.1 Sample collection

Fecal samples were collected from healthy young people (average age 24 years) in Inner Mongolia, Xinjiang, and Tibet (*n* = 38) (Table 1). All samples were collected with the consent of the volunteers themselves. Each sample comprised of 5~10 g of human feces, which were picked up in a sterile sampling bottle, marked, and sealed with a sealing film, then quickly placed in an ice box and transferred into a car refrigerator for storage at −4℃. The samples were transported back to the laboratory for bacterial isolation within 2 h. Reference probiotic strains *Lactobacillus plantarum* P8 and *Lactobacillus casei* Zhang were provided by the Lactic Acid Bacteria Culture Collection (LABCC) of the Key Laboratory of Dairy Biotechnology and Engineering of the Ministry of Education of Inner Mongolia Agricultural University.

**TABLE 1:** The kind of samples and collected site in Mongolia, Tibetan, and XinJiang

Sample	Gender	Region	Age
FHS12	Male	Inner Mongolia	21
FHS15	Male	Inner Mongolia	33
FHS16	Female	Inner Mongolia	22
FHS2	Male	Inner Mongolia	30
FHS4	Male	Inner Mongolia	35
FHS7	Female	Inner Mongolia	21
FHS8	Female	Inner Mongolia	21
FXJ21	Female	Xinjiang	22
FXJ26	Female	Xinjiang	23
FXJ27	Female	Xinjiang	22
FXJ28	Female	Xinjiang	20
FXJ36	Female	Xinjiang	28
FXJ38	Female	Xinjiang	18
FXJ4	Male	Xinjiang	21
FXJ40	Male	Xinjiang	24
FXJ47	Male	Xinjiang	21
FXJ5	Male	Xinjiang	23
FXJ56	Male	Xinjiang	26
FXJ6	Male	Xinjiang	22
FXM13	Female	Inner Mongolia	30
FXM19	Male	Inner Mongolia	29
FXM20	Female	Inner Mongolia	29
FXM22	Male	Inner Mongolia	33
FXM23	Female	Inner Mongolia	30
FXZ15	Female	Tibet	27
FXZ16	Female	Tibet	24
FXZ24	Male	Tibet	24
FXZ25	Male	Tibet	24
FXZ30	Male	Tibet	22
FXZ33	Female	Tibet	25
FXZ34	Female	Tibet	25
FXZ37	Female	Tibet	35
FXZ45	Female	Tibet	29
FXZ46	Male	Tibet	24
FXZ49	Female	Tibet	22
FXZ5	Female	Tibet	28
FXZ52	Female	Tibet	23
FXZ53	Male	Tibet	26

### 2.2 Isolation, purification, and preservation of *Lactobacillus*

Lactic acid bacteria were counted using the pouring culture method (Liu Hongxin et al., 2017). The stool sample (5 g) was diluted with normal saline (45 ml), and the appropriate concentration gradients (10^–5^, 10^–6^, and 10^–7^) were selected by the 10‐fold gradient dilution method (Yu et al., .2015). The diluent of 1 ml sample was absorbed and shaken in the unsolidified MRS solid medium, and 0.5% l‐cysteine and 0.05 mg/ml mupirocin (Qingdao Hope Biotechnology Co., Ltd., China) (Ferraris et al., 2010) were added to modify MRS Agar (Oxoid). After the culture medium was thoroughly dried, the dishes were incubated at 37℃ in an anaerobic environment for 48 h (Jousimies‐Sommer & Summanen, 2002). At the same time, 100 μL of sample dilution with the same dilution multiple as living bacteria count was spread on MRS solid medium. After colony formation, single colonies with different morphological characteristics were randomly selected on the corresponding solid medium for 2–3 times of lineation purification (Gooch Jan, 2011), in order to screen out colonies with different morphology. The single colony was stained with Gram, and the cell morphology was observed under a microscope. The pure isolate identified as gram‐positive was cultured in modified MRS solid medium. After catalase test, the catalase‐positive bacteria were discarded. Finally, the gram‐positive and catalase‐negative isolates were tentatively designated as lactic acid bacteria (Daiwen., 1999). Those tentatively designated as *Lactobacillus* were cultured at 37℃ in an anaerobic environment for 18–24 h and prepared for DNA extraction (see below) and preservation. After centrifugation at 3500 × g for 10 min, the supernatant was discarded and replaced with about 5 ml normal saline into which the bacteria in the precipitate were resuspended. This was repeated three times in more saline to wash the bacterial cells which were finally resuspended using a Pap needle in a sterile protective solution containing: 10 g skimmed milk powder, 0.1 g sodium glutamate, 90 ml distilled water, all sterilized at 121℃ for 7 min. Some of the isolates were stored in a cryopreservation tube at −80℃, and the other part was freeze‐dried in an ampoule tube and cryopreserved in a refrigerator at −80℃.

### 2.3 Sequence analysis of 16S rRNA gene

For extraction of DNA from each tentatively identified *Lactobacillus* strain, 2 ml of cells in liquid culture medium was centrifuged for 1 min at 12000 rpm. The supernatant was discarded, and the bacterial precipitate was collected. The genomic DNA extracted using DNA extraction kit (Beijing Tiangen Biotechnology Co., Ltd., China) needs to be determined by a ND‐1000 micro‐ultraviolet spectrophotometer. Fifty microliters of purified DNA was diluted to 100 ng/ μL with a automatic thermal cycler (MJ Research PTC‐200; MJ Research Inc., Watertown, MA, USA) for 16S rRNA amplification and sequencing. The primers used to amplify the 16S rRNA gene were universal primers, the forward primer FA‐27F (5'‐GCAGAGTTCTCGGA GTCACGAAGAGTTTGATCCTGGCTCAG‐3') and the reverse primer RA‐1495R (5'‐AGCGGA TCACTTCACA CAGGACTACGGCTACCTTGTTACGA‐3') (Sun et al., 2010). Each PCR reaction (50 μL) includes 1.5 μL (10 μmol/L) upstream and downstream primers, 2.0 μL genomic DNA, 5.0 μL 10 × buffer, 4 μL dNTP (2.5 mmol/L), 0.5 μL Taq DNA polymerase, and 35.5 μL distilled water. PCR amplification was performed under following conditions: pre‐denaturation at 94℃ for 5 min, 30 cycles (denaturation at 94℃, 58℃, annealing for 1 min, extension of 2 min at 72℃), and final extension at 72℃ for 10 min, and the product was kept at 4℃ to prevent degradation (Liu et al., 2020). After the amplification reaction, approximately 2 μL of the PCR amplification products was analyzed by 1% agarose gel electrophoresis and visualized on the gel imager (Beijing SAGE Creative Science Co., Ltd., Beijing, China). If there was a clear amplification band at 1500 bp and no trailing or diffusion phenomena, the PCR amplification was considered successful. Positive products were sent directly for sequencing by Majorbio Bio Pharm Technology Co., Ltd. (Shanghai, China). Sequences were compared using the SeqMan module of the DNA Star software (DNA‐STAR Company, Madison, Wisconsin, USA) (Altschul et al., 1997). A BLAST search (http://BLAST.ncbi.nlm.nih.gov/BLAST.cgi) of the NCBI database was used to analyze the nucleic acid sequences of bacteria in relation to sequences in GenBank. When sequence similarity to GenBank sequences achieved 97%, they were considered to be the same genus; when sequence similarity reached 99%, they were determined to be the same species (Drancourt et al., 2000). Finally, MEGA software version 6.0 was used build the system evolution tree (Tamura et al., 2007; Yu et al., 2011).

### 2.4 Physiological and biochemical evaluation

#### 2.4.1 In vitro growth curve

Cryopreserved isolate was inoculated into modified MRS liquid medium, incubated at 37℃ for 24 h, and then continuously cultured for three generations to activate them (Yasmin et al., 2020). Inoculum (4%) was inoculated into MRS liquid medium, and there was MRS liquid medium 4.5 ml into each test tube (15 × 100 mm). The experiment was divided into three groups, with 12 replicative test tubes in each group, which were cultured at 37℃ for 24 h. Samples (three parallel samples) were taken every 2 h and stored in a refrigerator at 4℃. After sampling, the MRS liquid medium was used as blank, the absorbance value of the sample was determined using a spectrophotometer under 620 nm, and the growth curve was drawn with culture time as abscissa and corresponding absorbance value as ordinate.

### 2.5 Tolerance to acid environments

#### 2.5.1 Preparation of artificial gastric and intestinal fluids

Artificial gastric juice was prepared using 3.0 g/L pepsin dissolved in sterile phosphate‐buffered saline (PBS; China Beijing Chemical Base Science and Technology Co., Ltd.) that had been adjusted to pH 2.5 with 0.1 mol/L hydrochloric acid. The resulting artificial gastric fluid was then filtered through a 0.22‐μm filter membrane (China Hefei Biosharp Biotechnology Co., Ltd.) to sterilize it prior to use. Artificial intestinal fluid was prepared using 1.0 g/L trypsin (China Beijing Chemical Base Technology Co., Ltd.) and 1.8% bile salt (China Beijing Chemical Base Technology Co., Ltd.) dissolved in sterile PBS and adjusted to pH 8.0 with 0.1 mol/L sodium hydroxide. The resulting artificial intestinal fluid was filtered through a 0.22‐μm filter membrane (China Hefei Biochemical Co., Ltd.) to sterilize it prior to use.

### 2.6 Tolerance to artificial digestive juices

Cryopreserved Isolated *Lactobacillus* strains were activated as described previously. Activated isolate was centrifuged (3800 × g) for 10 min and the supernatant removed. The bacterial pellets were collected and washed three times in PBS (8.0 g/L NaCl, 0.2 g/L KH_2_PO_4,_ and 1.15 g/L Na_2_HPO_4_, pH 7.2). The bacteria were then resuspended in 5 ml of sterilized PBS. For each strain, 0.5 ml of bacterial suspension was added to 4.5 ml (pH 2.5) of artificial gastric fluid and then incubated at 37℃ under anaerobic conditions. Samples were taken at 0 h and 3 h, respectively, and plate viable bacteria were counted (Fernández et al., 2003). The artificial gastric juice cultured with pH 2.5 for 3 h was transferred into artificial intestinal juice (4.5 ml) by absorbing 0.5 ml, and then anaerobic culture was performed. The number of living bacteria was determined after 4 h and 8 h (Hosono, 1998). Finally, the survival rate was calculated using the plate counting method (Bao et al., 2010). Specifically, strain survival rate = [N_1_/N_0_] × 100%, where N_0_ is the number of live bacteria at 0 h [colony forming units (CFU/ml)] and N_1_ is the number of live bacteria in artificial gastric/intestinal fluids after 3 h or 11 h (CFU/ml).

### 2.7 Bile salt tolerance

Each of the cryopreserved *Lactobacillus* strains was activated as described previously. The activated *Lactobacillus* was inoculated in MRS‐sodium mercaptoacetate medium (MRS liquid medium added 0.2% sodium mercaptoacetate) without bovine bile salt and MRS‐sodium mercaptoacetate solution containing bile salt (3.0 g/L) according to 4% inoculum, and then cultured at 37℃ in constant temperature and anaerobic culture. The OD values of cultures were determined at 620 nm every 1 h by an UV‐vis spectrophotometer (Kyoto Shimadzu Company of Japan). When the OD value of cultures had increased by more than 0.3 U, the experiment was stopped, and the time required for each strain to achieve 0.3 U was recorded. The delay time (LT) was calculated as the difference between values with and without bile salts (Pochart et al., 1992). The tolerance of isolate to bile salt was judged according to the delay time of each strain.

### 2.8 Fermentation and storage characteristics

Skimmed milk (10% in distilled water plus 1% glucose) was sterilized and cooled to about 40 ~ 45℃. For each strain, 4.5 ml of skimmed milk in a test tube was inoculated with 0.5 ml of a 1.0 × 10^7^ CFU/ml suspension of activated bacteria, shaken well, and incubated at 37℃ under anaerobic conditions. The pH and titrated acidity were measured every 3 h until the pH value reached 3.5. At the end of fermentation, the resulting fermented milk was stored at 4℃, and the number of viable bacteria, pH, and titratable acidity were determined after storage for 0, 7, 14, and 21 days, respectively. Titratable acidity of the fermented milk was determined according to the national standard method reference GB5413‐34–2010 (National Standard of the People's Republic of China, 2010).

### 2.9 Statistical analysis

Three parallel samples were made for the determination of each index of the test sample. The experimental data were statistically analyzed by the CORR program of SAS9.0 software, and the drawing was made by Origin7.0 software.

## 3 RESULTS AND DISCUSSION

### 3.1 Preliminary identification of *Lactobacillus*

Continuous culture of isolates in modified MRS solid medium for 48 h resulted in colonies that were white, transparent, with neat edges, smooth or dull surfaces, flattened, or protruding in the center. By Gram staining and other phenotypic tests, a total of 57 isolates were identified as *Lactobacillus* species. All isolates showed gram‐positive and catalase‐negative properties.

### 3.2 Molecular identification by 16S rRNA sequencing

**FIGURE 1:**
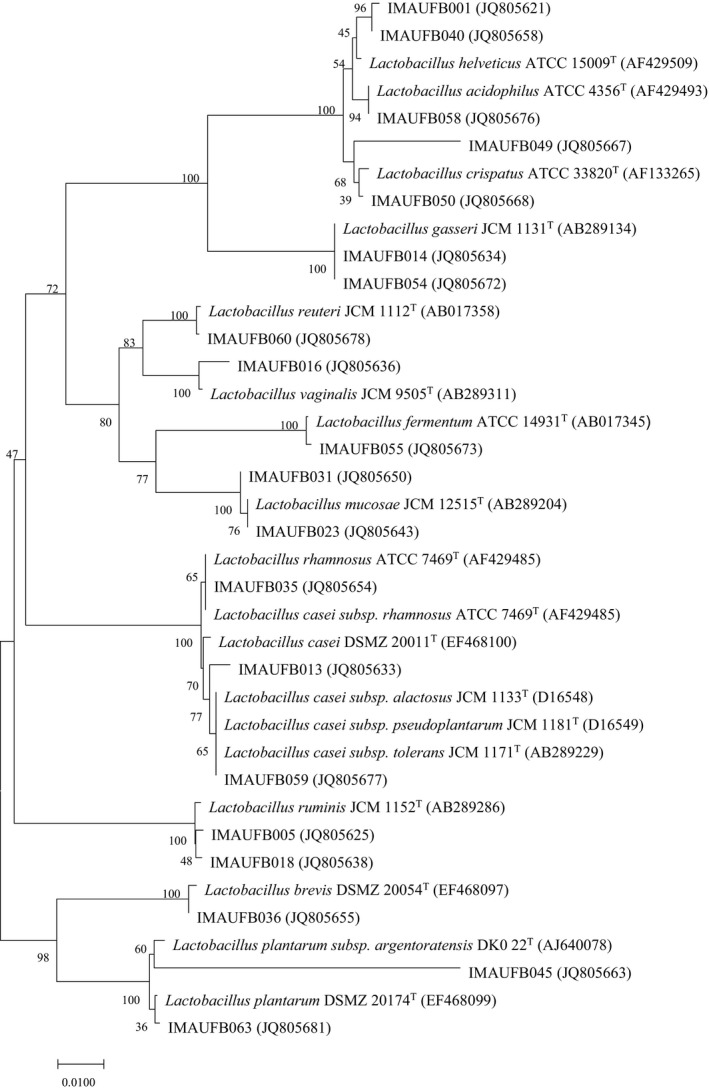
Phylogenetic tree of *Lactobacillus* isolates from the human intestine based on 16S rRNA

The 57 isolates of *Lactobacillus* from human feces could be divided into 13 species by 16S rRNA gene sequencing (Figure 1), specifically: *L*. *ruminis* (31 isolates), *L. gasseri* (8 isolates), *L. casei*, *L. vaginalis*, *L. mucosae*, *L. brevis*, *L. helveticus*, *L. crispatus*, *L. acidophilu*, *L. reuteri*, *L. plantarum*, *L. fermentum,* and *L. rhamnosus*. The species *L. ruminus* and *L. gasseri* were the dominant species of *Lactobacillus* in the human intestinal tract in this study. Specifically, *L. ruminus* accounted for 54.38% of the total number of isolated bacteria while *L. gasseri* accounted for 14.03%.

A total of 23 isolates of *Lactobacillus* were isolated from samples collected in Tibet; 15 of these were *L. ruminus* accounting for 65.21% of all bacteria from the fecal samples of young people in Tibet (Table 2). Of the remaining isolates from Tibet samples, three were *L. gasseri* and there were one each of *L. helveticus*, *L. crispatus*, *L. plantarum*, *L. casei,* and *L. vaginalis*. Thus, *L. ruminus* and *L. gasseri* were the dominant *Lactobacillus* species in the fecal samples of young people in Tibet. A total of 18 isolates of *Lactobacillus* from seven species were isolated from fecal samples collected in Xinjiang: *L*. *ruminus* (10 isolates), *L. mucosae* (3 isolates), and one strain each of *L. gasseri*, *L. crispatus, L. plantarum, L. rhamnosus,* and *L. brevis*. Thus, *L. ruminus* and *L. mucosae* were the dominant *Lactobacillus* species in the fecal samples of young people in Xinjiang. A total of 16 isolates of *Lactobacillus* from eight species were isolated from fecal samples collected in Inner Mongolia: *L*. *ruminus* (6 isolates), *L. gasseri* (4 isolates), and one strain each of *L. helveticus*, *L. casei*, *L. acidophilus*, *L. reuteri*, and *L. crispatus*. Thus, *L. ruminus* and *L. gasseri* were the dominant *Lactobacillus* species in the fecal samples of young people in Inner Mongolia.

**TABLE 2:** Number of *Lactobacillus* species isolated from human feces collected in different regions of China

Species	Tibet	Xinjiang	Inner Mongolia	Total
*L. ruminus*	15	10	6	31
*L. gasseri*	3	1	4	8
*L. helveticus*	1	0	1	2
*L. casei*	1	0	1	2
*L. vaginalis*	1	0	0	1
*L. mucosae*	0	3	0	3
*L. rhamnosus*	0	1	0	1
*L. brevis*	0	1	0	1
*L. acidophilus*	0	0	1	1
*L. reuteri*	0	0	1	1
*L. fermentum*	0	0	1	1
*L. crispatus*	1	1	1	3
*L. plantarum*	1	1	0	2
Total	23	18	16	57

The dominant *Lactobacillus* species in the three regions were similar; *L. ruminus* was the predominant *Lactobacillus* in all three regions. Overall, *L. ruminus* and *L. gasseri* were the main *Lactobacillus* microflora in the intestinal tract of healthy young people in Tibet, Xinjiang, and Inner Mongolia. This was consistent with the results of studies of fecal samples from young French people (Heilig et al., 2002) and young European (Klaenhammer, 1995) who also found that *Lactobacillus* species in the human intestinal tract were mainly comprised of *L. gasseri* and *L. reuteri*. However, it was different from a study on the dominant *Lactobacillus* species in fecal samples from 20‐year‐old and 25‐year‐old Chinese people which found *Lactobacillus curly* and *Lactobacillus saliva* are common and dominant *Lactobacillus* in the intestinal tract of healthy Chinese young people, and the species and quantity of other *Lactobacillus* are also quite different from those of Europeans (Min et al., 2004). Many studies have found significant differences in diversity and abundance of *Lactobacillus* among individual and between regions (Liu Hongxin et al., 2017). The fact that the dominant *Lactobacillus* species from young people in Tibet, Xinjiang, and Inner Mongolia detected in this experiment was similar to that of Europeans may be because the diet of these three regions is similar to that of Europeans, that is, a lot of dairy products.

### 3.3 In vitro growth curve

**FIGURE 2:**
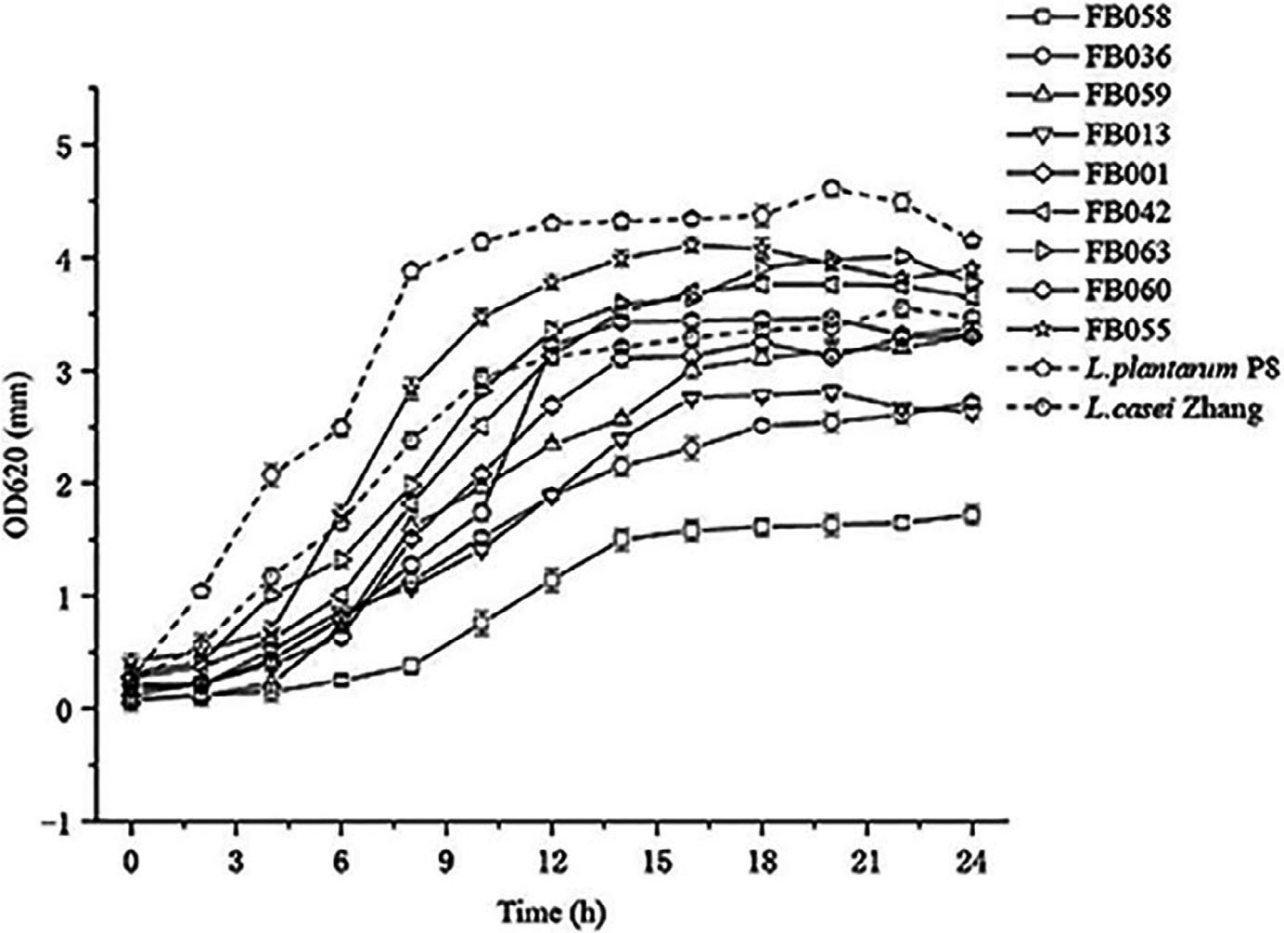
Growth of different *Lactobacillus* isolates in MRS at 37℃

We evaluated the growth of nine of the *Lactobacillus* (from seven species) isolated from fecal samples and two reference strains: *L*. *plantarum* P8 and *L. casei* Zhang. It can be seen from the Figure 2 that the growth curves of nine isolates of *Lactobacillus* isolated in MRS liquid medium were basically very similar to each other. They all grew slowly between 0 and 4 h, entered a stable growth period after 14 h which lasted until 24 h. In this experiment, growth characteristics were evaluated under aerobic conditions and so differences in growth were also affected by oxygen. Thus, in subsequent experiments we adjusted the culture time according to the growth curve.

### 3.4 Acid and bile tolerance

Preliminary analysis of acid tolerance (pH 2.0) and artificial gastric juice tolerance (pH 3.0) of 11 *Lactobacillus* can be seen in Table 2. All the tested *Lactobacillus* could grow well in the pH 3.0 environment, but were unable to survive in the pH 2.0 environment for very long. This is consistent with the results of Marteau (Marteau et al., 1997). After 3 h at pH 3.0 in artificial gastric juice, survival rates of the different isolates of *Lactobacillus* varied significantly from that of the two reference strains (*p* < .05) (Table 3). From the experimental results, it can be seen that the survival rate of the isolated *L*. *plantarum* IMAUFB042, IMAUFB063, and the control strain *L*. *plantarum* P8 in pH 3.0 gastric juice environment reached more than 90%, showing a strong tolerance to artificial gastric juice, which is consistent with the conclusion that part of *Lactobacillus* isolated from human body has good resistance to gastrointestinal fluid, which was found by Fernández, Boris, and Barbés (2003). In other studies, it was also found that *L*. *plantarum* had strong tolerance to artificial gastric juice with low pH value (Peng et al. 2010; Wang et al. 2004).

**TABLE 3:** Tolerance of *Lactobacillus* to artificial gastrointestinal juices (pH 3.0) and survival rates after 0 h and 3 h incubation (*n* = 3, x ± *SD*)

Isolate reference number	Mean tolerance		Survival rate (%)
	0 h	3 h	
*L. acidophilus* IMAUFB058	9.13 ± 0.01	8.91 ± 0.02	60.0 ^h^
*L. brevis* IMAUFB036	9.09 ± 0.02	8.95 ± 0.01	71.1 ^f^
*L. casei* IMAUFB059	9.04 ± 0.01	8.79 ± 0.03	58.1 ^i^
*L. casei* IMAUFB013	8.98 ± 0.03	8.85 ± 0.01	73.9 ^e^
*L. casei* Zhang	8.97 ± 0.01	8.78 ± 0.05	65.3 ^g^
*L. helveticus* IMAUFB001	9.16 ± 0.03	9.09 ± 0.04	87.4 ^d^
*L. plantarum* IMAUFB042	8.31 ± 0.02	8.43 ± 0.06	100 ^a^
*L. plantarum* IMAUFB063	8.99 ± 0.01	9.11 ± 0.05	100 ^a^
*L. plantarum* P8	8.80 ± 0.04	8.87 ± 0.05	97.1 ^b^
*L. reuteri* IMAUFB060	9.13 ± 0.01	8.01 ± 0.06	7.5 ^j^
*L. fermentum* IMAUFB055	9.25 ± 0.03	9.22 ± 0.01	94.4 ^c^

Note

Presented values are means of triplicate determinations; ± indicates standard deviation from the mean. Data in the same column followed by different lowercase letter are significantly different from each other (*p* < .05).

**FIGURE 3:**
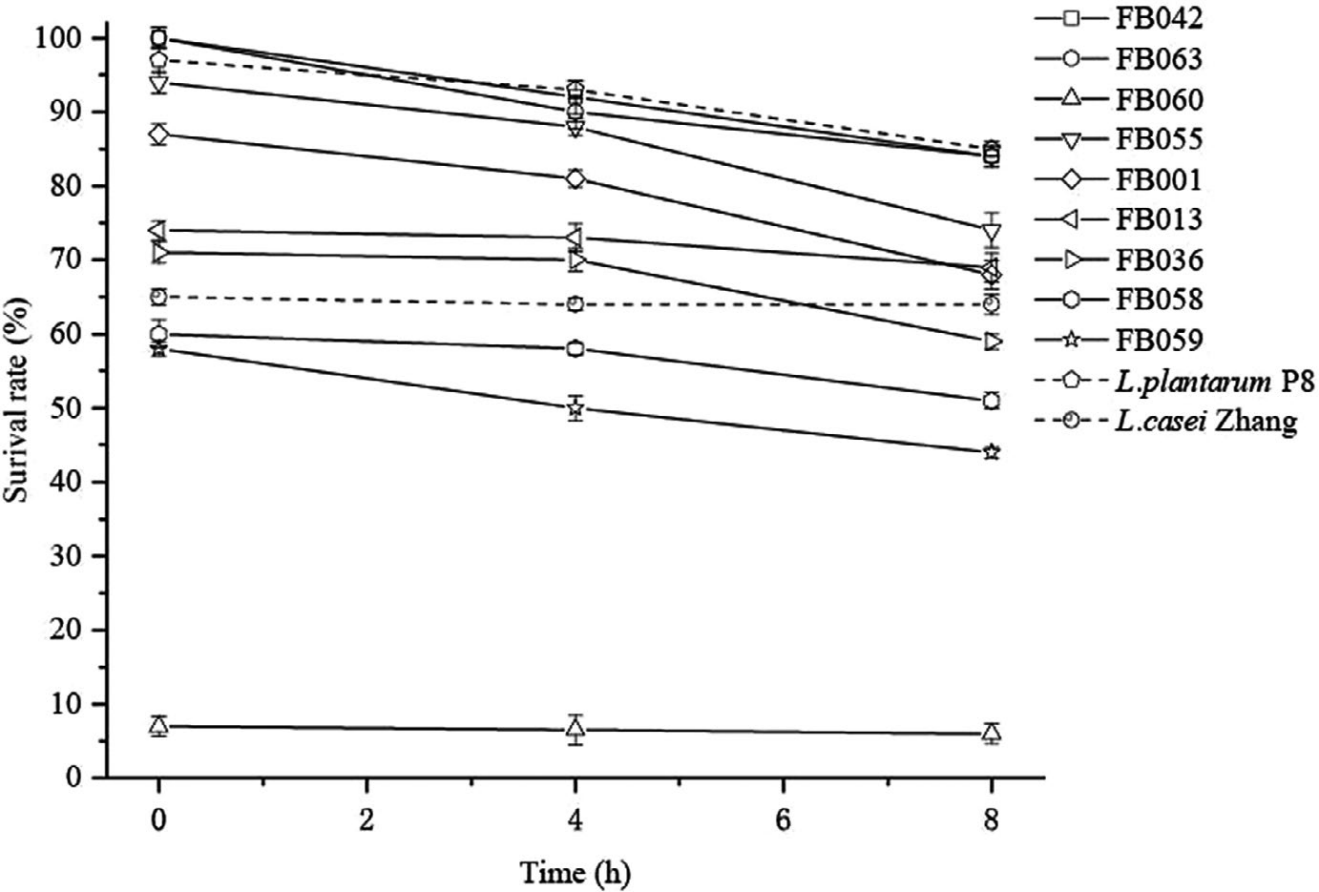
Survival rates of *Lactobacillus* isolates in artificial intestinal juice at pH 8.0

The 11 isolates of *Lactobacillus* that were digested at pH 3.0 in artificial gastric juice for 3 h were then inoculated into artificial intestinal juice at pH 8.0 and their survival rate was recorded after 4 h and 8 h (Figure 3). The number of *Lactobacillus* viable count changed slowly during 4 h and 8 h digestion in pH 8.0 artificial intestinal juice, but the tolerance of them is becoming different after 8 h digestion (*p* < .05). Among them, *L. plantarum* IMAUFB042, IMAUFB063, and the control strain *L. plantarum* P8 maintained a high survival rate of 80% after 8 h, but the survival rate of *L. reuteri* IMAUFB060 was very low after this period of time, and significantly lower than the other *Lactobacillus* (*p* < .05).  

Bile concentration in the human intestinal tract generally fluctuates between 0.3% and 1.8%. Of the 11 *Lactobacillus* evaluated, 10 were tolerant to bile salts in the range of 0.3% ‐ 2.0% (Table 4). The highest bile salt concentration that *L. helveticus* IMAUFB001 could tolerate was 1.8%. Overall, some of the isolated strains of *Lactobacillus* we evaluated demonstrated high resistance to bile salts which was consistent with previous research showing that most intestinal bacteria have strong tolerance to bile salts (Gilliland & Walker, 1990). Tolerance in the reference strains *L. casei* Zhang and *L. plantarum* P8 to 1.8% bile salts was consistent with previous studies on these isolates (Wu et al., 2009; Ying et al., 2007). Another strain of the same species, *L. plantarum* MB3182, also has good tolerance to 2.0% bile salt (Missotten et al., 2008), confirming the conclusion that *L. plantarum* has good bile salt tolerance.

**TABLE 4:** Tolerance of *Lactobacillus* to different concentrations bile salts

Isolate reference number		Bile salt concentration(%)						
		0.3	0.6	0.8	1.0	1.5	1.8	2.0
*L. acidophilus* IMAUFB058	△A_620nm_	2.593	1.981	1.615	1.411	1.153	0.661	0.31
	MRS plate	﹢	﹢	﹢	﹢	﹢	﹢	﹢
*L. brevis* IMAUFB036	△A_620nm_	2.834	1.865	1.541	1.277	1.193	0.875	0.728
	MRS plate	﹢	﹢	﹢	﹢	﹢	﹢	﹢
*L. casei* IMAUFB059	△A_620nm_	1.488	1.068	0.756	0.816	0.792	1.128	0.975
	MRS plate	﹢	﹢	﹢	﹢	﹢	﹢	﹢
*L. casei* IMAUFB013	△A_620nm_	3.217	1.849	1.837	1.597	1.711	1.643	1.003
	MRS plate	﹢	﹢	﹢	﹢	﹢	﹢	﹢
*L. casei Zhang*	△A_620nm_	2.842	1.768	1.936	0.82	0.976	0.636	0.544
	MRS plate	﹢	﹢	﹢	﹢	﹢	﹢	﹢
*L. helveticus*IMAUFB001	△A_620nm_	2.814	1.211	1.914	0.968	0.573	0.424	‐
	MRS plate	﹢	﹢	﹢	﹢	﹢	‐	‐
*L. plantarum* IMAUFB042	△A_620nm_	0.738	0.387	0.369	0.384	0.426	0.534	0.66
	MRS plate	﹢	﹢	﹢	﹢	﹢	﹢	﹢
*L. plantarum* IMAUFB063	△A_620nm_	1.114	0.637	0.589	0.466	0.475	0.484	0.61
	MRS plate	﹢	﹢	﹢	﹢	﹢	﹢	﹢
*L. plantarum* P8	△A_620nm_	3.118	1.778	1.676	1.502	1.244	0.338	0.38
	MRS plate	﹢	﹢	﹢	﹢	﹢	﹢	﹢
*L. reuteri*IMAUFB060	△A_620nm_	1.913	1.745	1.001	1.469	0.965	0.877	0.952
	MRS plate	﹢	﹢	﹢	﹢	﹢	﹢	﹢
*L. fermentum* IMAUFB055	△A_620nm_	1.24	0.838	0.583	0.934	0.405	0.371	0.323
	MRS plate	﹢	﹢	﹢	﹢	﹢	﹢	﹢

Note

△A620nm is the change of absorbance value for each isolate within 12 h; + indicates colony growth, ‐indicates no colony growth.

The delay time of each strain in response to bile salts was significantly different among isolates (*p* < .05), indicating significant differences in bile salt tolerance among isolate (Table 5). The delay time of the reference strain *L. casei* Zhang was 0.65 h, which was similar to the delay times of *L. acidophilus* IMAUFB058, *L. brevis* IMAUFB036, and *L. casei* IMAUFB059 which were 0.78 h, 0.87 h, and 0.91 h, respectively; the delay time of *L. helveticus* IMAUFB001, which had good tolerance to bile salts, was more than 5 h, which was significantly different from all the other isolates (*p* < .05). While Liong & Shah, 2005 showed that probiotics in general varied significantly in bile tolerance, the *Lactobacillus,* we evaluated strong tolerance to bile salt which is likely related to the long‐term maintenance of high bile salt concentration in the intestinal environment from which they originated.

**TABLE 5:** Comparison of growth rate and delay time of *Lactobacillus* at 3% bile salts concentration (*n* = 3, x ± *SD*)

Isolate reference number	OD_620nm_ The time required to increase the number of units by 0.3 (h)		
	Mean time in medium containing 0.3% bile salt	Mean time in bile‐free medium	Delay
*L.acidophilus* IMAUFB058	3.38 ± 0.23	2.60 ± 0.15	0.78 ± 0.08 ^ef^
*L. brevis* IMAUFB036	3.11 ± 0.21	2.24 ± 0.19	0.87 ± 0.02 ^def^
*L. casei* IMAUFB059	4.08 ± 0.23	3.17 ± 0.20	0.91 ± 0.03 ^de^
*L. casei* IMAUFB013	4.17 ± 0.11	3.15 ± 0.02	1.02 ± 0.09 ^bcd^
*L. casei* Zhang	3.70 ± 0.16	3.05 ± 0.17	0.65 ± 0.01^f^
*L. helveticus* IMAUFB001	8.89 ± 0.34	3.32 ± 0.08	5.57 ± 0.26 ^a^
*L. plantarum* IMAUFB042	3.84 ± 0.11	2.86 ± 0.02	0.98 ± 0.09 ^bcd^
*L. plantarum* IMAUFB063	3.22 ± 0.21	2.08 ± 0.09	1.14 ± 0.13^bc^
*L. plantarum* P8	3.73 ± 0.24	2.55 ± 0.18	1.18 ± 0.06 ^b^
*L. reuteri* IMAUFB060	4.46 ± 0.13	3.33 ± 0.32	1.13 ± 0.19 ^bc^
*L. fermentum* IMAUFB055	2.96 ± 0.32	1.74 ± 0.39	0.95 ± 0.07 ^cde^

Note

Presented values are means of triplicate determinations; ± indicates standard deviation from the mean. Data in the same column followed by different lowercase letter are significantly different from each other (*p* < .05).

**FIGURE 4:**
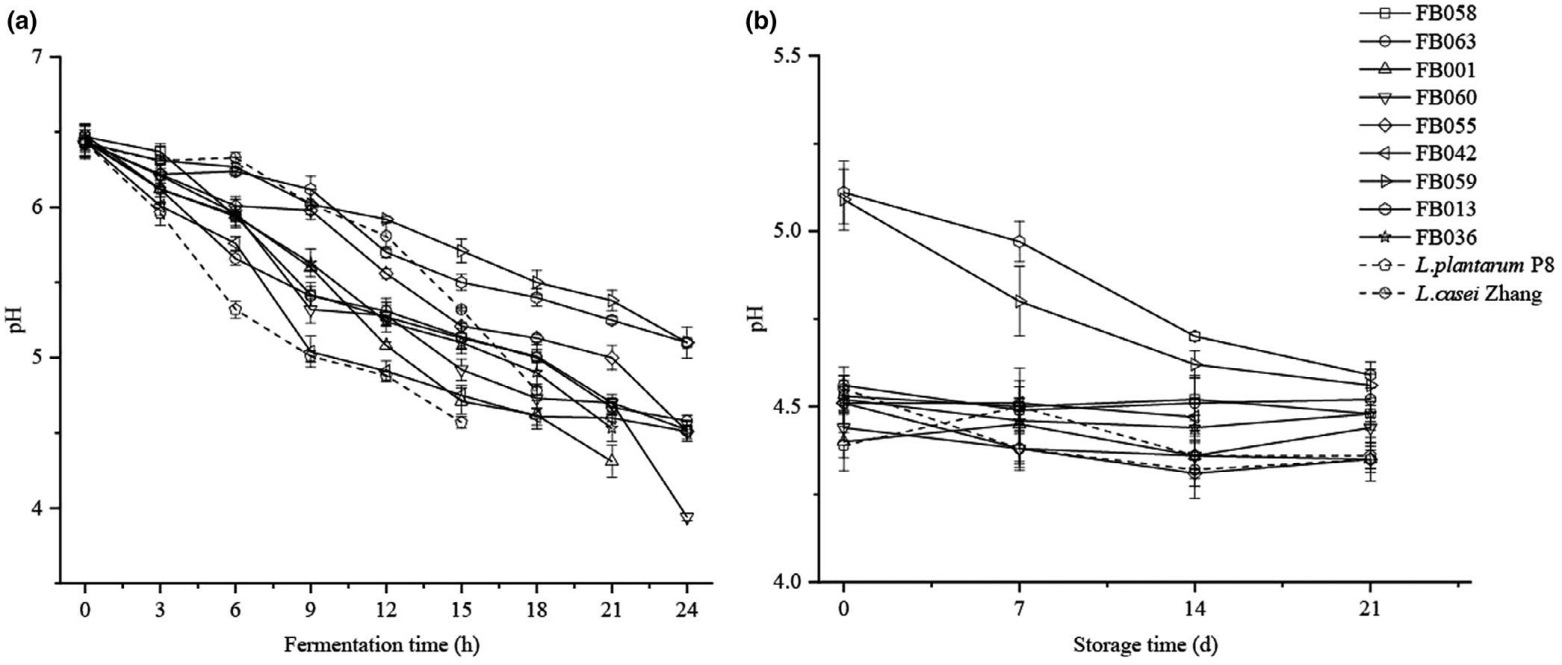
Changes of pH during fermentation (a) and storage (b) of yogurt by different *Lactobacillus* isolates

The pH value of each strain decreased with fermentation time; the decrease was slow in the first 3 h of fermentation, and then accelerated (Figure 4). Fermentation by most of the *Lactobacillus* was strong, reducing the pH to 4.5 within 22 h, while the ability of *L. casei* IMAUFB059 and IMAUFB013 to ferment skimmed milk was weak and the pH achieved during fermentation was not as low as for the other *Lactobacillus*. The pH of each strain changed slowly during storage because of growth, and therefore, acid production was suppressed at 4℃. While the pH of *L. casei* IMAUFB059 and IMAUFB013 did continue to decrease during storage, it was still higher than 4.5 after 21 days. Overall, acid production during fermentation was lower for *Lactobacillus* than for *L. plantarum* P8 and *L. casei* Zhang.

**FIGURE 5:**
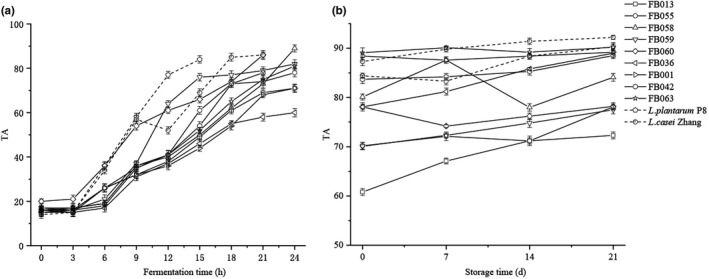
Changes in titratable acidity during fermentation (a) and storage (b) of yogurt by different *Lactobacillus* isolates

During the fermentation of skimmed milk, the titration acidity of each strain increased significantly with the fermentation time. The changing trend of titration acidity between 0 and 3 h is slow, the titration acidity is lower, and the titration acidity increases rapidly between 3 and 18 h (Figure 5). At the end of fermentation, the TA values of the nine isolates of *Lactobacillus* were lower than those of the two reference strains, *L. plantarum* P8 and *L. casei* Zhang. The significant increase in acidity during fermentation by *Lactobacillus* is mainly due to the fact that *Lactobacillus* ferments lactose in skimmed milk into organic acids (mainly lactic acid); the continuous accumulation of organic acids leads to the continuous increase in acidity. The upward trend in titratable acidity during fermentation slowed during cold storage at 4℃. After 21 days of storage, the TA value of *Lactobacillus* was still higher than that of *L. plantarum* P8 and *L. casei* Zhang.

After 21 days of storage, the number of living bacteria in the fermented milk had decreased by about 3 logarithmic units (Table 6). This is consistent with other studies that found the number of living bacteria of some probiotic decreased to a certain extent during low‐temperature storage of fermented milk (Kailasapathy, 2006; Shah, 2000). While the number of living bacteria in fermented milk produced by some isolates remained above 1.0 × 10^7^ CFU/ml after 21 days of storage, the number of living bacteria of the reference strain, *L. casei* Zhang, after 21 days of storage was slightly higher than for the *Lactobacillus* isolated from human feces and also the other reference strain, *L. plantarum* P8. This indicates that the storage characteristics of *L. casei* Zhang‐fermented milk were better than for some of the *Lactobacillus* isolated from the intestinal tract.

**TABLE 6:** Change in viable counts of different *Lactobacillus* strains in yogurt after 21 d storage at 4℃ (*n* = 3, x ± *SD*)

Isolate reference number	0 d	7 d	14 d	21 d
*L. acidophilus* IMAUFB058	9.29 ± 0.02	8.76 ± 0.03	7.89 ± 0.07	7.17 ± 0.02
*L. brevis* IMAUFB036	9.19 ± 0.02	8.57 ± 0.1	8.14 ± 0.09	8.12 ± 0.08
*L. casei* IMAUFB059	9.14 ± 0.04	8.94 ± 0.05	7.79 ± 0.03	7.05 ± 0.04
*L. casei* IMAUFB013	9.23 ± 0.09	8.62 ± 0.06	7.19 ± 0.11	7.03 ± 0.1
*L. casei* Zhang	9.08 ± 0.01	8.76 ± 0.03	8.63 ± 0.06	8.16 ± 0.04
*L. helveticus* IMAUFB001	9.18 ± 0.02	8.54 ± 0.03	7.78 ± 0.19	7.76 ± 0.03
*L. plantarum* IMAUFB042	9.31 ± 0.08	8.79 ± 0.06	8.63 ± 0.09	7.54 ± 0.06
*L. plantarum* IMAUFB063	9.02 ± 0.14	8.1 ± 0.02	8.19 ± 0.06	7.05 ± 0.1
*L. plantarum* P8	9.37 ± 0.02	8.57 ± 0.04	8.08 ± 0.22	7.69 ± 0.07
*L. reuteri* IMAUFB060	9.13 ± 0.03	8.41 ± 0.08	7.88 ± 0.05	7.11 ± 0.08
*L. fermentum* IMAUFB055	9.28 ± 0.12	8.41 ± 0.13	8.14 ± 0.09	7.89 ± 0.07

Note

Presented values are means of triplicate determinations; ± indicates standard deviation from the mean. Data in the same column followed by different lowercase letter are significantly different from each other (*p* < .05).

## 4 CONCLUSION

The fecal samples of healthy young individuals in Xinjiang, Tibet, and Inner Mongolia were analyzed using the pure culture method and 16S rRNA gene homology analysis. Fifty‐seven isolates of *Lactobacillus* were identified and preserved. These species had a rich diversity, in which *L. ruminus* and *L. gasseri* were the predominant species. Nine of the *Lactobacillus* isolates and two reference strains of *Lactobacillus* had high survival rate in a pH 3.0 environment. *L*. *plantarum* and the reference strain *L. plantarum* P8 showed high tolerance in artificial gastrointestinal juice. Six of *Lactobacillus* and reference strains were tolerant to bile at a concentration of 2.0%. The fermentation of 11 isolates of *Lactobacillus* was slow, but they had good storage characteristics. This study is of practical significance; understanding the growth fermentation characteristics and tolerance of *Lactobacillus* isolates from the human intestinal tract may provide valuable isolate for use as probiotics.

## AUTHOR CONTRIBUTIONS

**Chen Liu:** Methodology (equal); Resources (equal); Software (equal); Validation (equal); Visualization (equal); Writing‐original draft (supporting). **Fei Han:** Resources (equal); Software (equal); Visualization (equal); Writing‐original draft (supporting); Writing‐review & editing (equal). **LING CONG LIN:** Resources (supporting); Validation (equal); Visualization (supporting); Writing‐review & editing (equal). **Ting Sun:** Investigation (supporting); Resources (supporting); Software (supporting); Writing‐review & editing (supporting). **Bilege Menghe:** Resources (supporting); Supervision (supporting); Writing‐review & editing (supporting). **Wunjun Liu:** Resources (equal); Supervision (equal); Writing‐review & editing (equal).

## FUND PROJECT SUPPORT

The study was funded by National Key R&D Program (Project Code: 2018YFE0123500) Project Name: Study on the nutritional status of Mongolian population and the diversity of *Lactobacillus* in the intestinal tract—Bilege Menghe; Inner Mongolia Agricultural University Science and Technology Plan Project of School of Food Science and Engineering (Project Code: SPKJ202007)—Wenjun Liu; Inner Mongolia Autonomous Region Science and Technology Plan Project (Project Code: 2021GG0080)—Wenjun Liu.

## DATA AVAILABILITY STATEMENT

The data used to support the findings of this study are included in the article.
